# Biological and clinical implications of *FGFR* aberrations in paediatric and young adult cancers

**DOI:** 10.1038/s41388-023-02705-7

**Published:** 2023-05-02

**Authors:** Lauren M. Brown, Paul G. Ekert, Emmy D. G. Fleuren

**Affiliations:** 1 Children’s Cancer Institute, Lowy Cancer Research Centre, UNSW Sydney, Sydney, NSW Australia; 2 School of Clinical Medicine, UNSW Medicine & Health, UNSW Sydney, Sydney, NSW Australia; 3 University of New South Wales Centre for Childhood Cancer Research, UNSW Sydney, Sydney, NSW Australia; 4 Cancer Immunology Program, Peter MacCallum Cancer Centre, Parkville, VIC Australia

**Keywords:** Paediatric cancer, Oncogenes, Growth factor signalling

## Abstract

Rare but recurrent mutations in the fibroblast growth factor receptor (FGFR) pathways, most commonly in one of the four FGFR receptor tyrosine kinase genes, can potentially be targeted with broad-spectrum multi-kinase or FGFR selective inhibitors. The complete spectrum of these mutations in paediatric cancers is emerging as precision medicine programs perform comprehensive sequencing of individual tumours. Identification of patients most likely to benefit from FGFR inhibition currently rests on identifying activating FGFR mutations, gene fusions, or gene amplification events. However, the expanding use of transcriptome sequencing (RNAseq) has identified that many tumours overexpress FGFRs, in the absence of any genomic aberration. The challenge now presented is to determine when this indicates true FGFR oncogenic activity. Under-appreciated mechanisms of FGFR pathway activation, including alternate FGFR transcript expression and concomitant FGFR and FGF ligand expression, may mark those tumours where FGFR overexpression is indicative of a dependence on FGFR signalling. In this review, we provide a comprehensive and mechanistic overview of FGFR pathway aberrations and their functional consequences in paediatric cancer. We explore how FGFR over expression might be associated with true receptor activation. Further, we discuss the therapeutic implications of these aberrations in the paediatric setting and outline current and emerging therapeutic strategies to treat paediatric patients with FGFR-driven cancers.

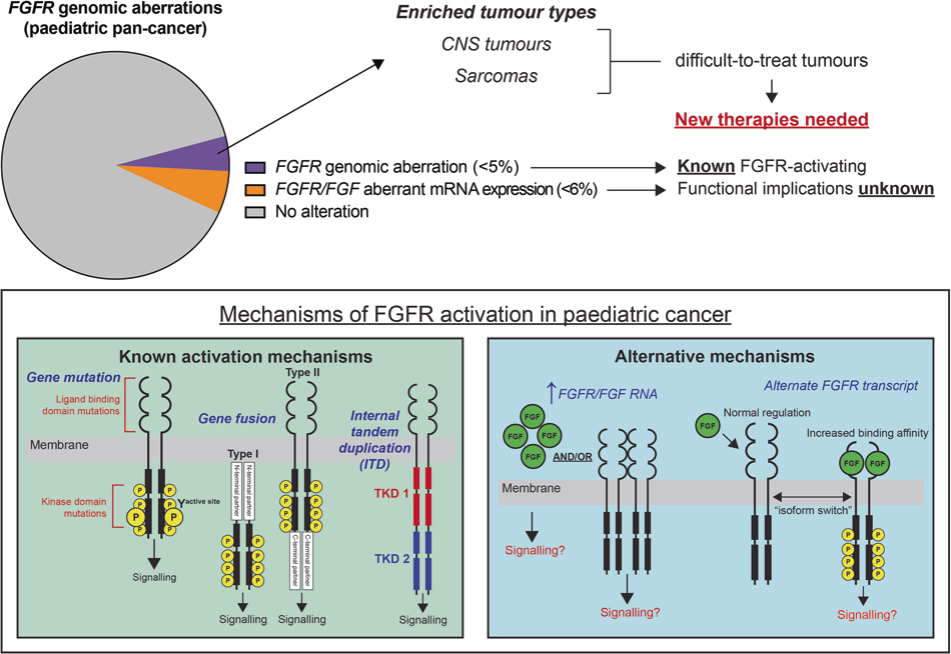

## Introduction

Receptor tyrosine kinases (RTKs) are cell surface receptors containing intracellular kinase domains. They regulate fundamental cellular processes including cell survival, proliferation, differentiation, and metabolism [1]. Large-scale genomic studies have shown that many kinase genes, including those that encode RTKs and non-receptor tyrosine kinases (NRTKs), are recurrently altered in cancer [2], and biological studies have characterised many as bona fide cancer driver genes. The most notable example in paediatric cancers being the *BCR-ABL1* fusion gene in B-cell acute lymphoblastic leukaemia (B-ALL) [3]. Strikingly, many other kinase-activating fusions that phenocopy BCR-ABL1 are now recognised in B-ALL, and characterise the new Ph-like B-ALL entity [4]. The importance of establishing the oncogenicity of tyrosine kinase (TK) driver genes is exemplified by the remarkable clinical success of ABL-targeted multi-kinase inhibitors, including imatinib [5] and dasatinib [6], in *BCR-ABL1* + and Ph-like B-ALL. Another remarkable success in this field is the NTRK-inhibitor larotrectinib, which has demonstrated clinical efficacy in a range of adult and paediatric cancers specifically driven by *NTRK* fusion genes [7]. There are, however, many more dysregulated TKs and TK pathways that represent potential drivers and novel drug targets in paediatric cancer that warrant further investigation. One that deserves further attention is the Fibroblast Growth Factor Receptor (FGFR) pathway.

An increasing body of data shows that aberrations in *FGFR* genes are present in many paediatric cancer types. The common *FGFR* genomic alterations include gene amplifications, gene mutations or single nucleotide variants (SNVs), and structural variants (SVs), which promote stemness, proliferation, angiogenesis, epithelial-mesenchymal transition, invasion and drug resistance in cancer cells [8]. Understanding whether *FGFR* RNA overexpression, even in the absence of genomic alteration, represents another important but less well understood mechanism of oncogenesis has the potential to expand the therapeutic reach of FGFR inhibitors [9].

Pan-cancer genomic studies and precision medicine clinical trials have enabled greater understanding of the prevalence of *FGFR* pathway aberrations in paediatric cancer [10–16]. These studies have demonstrated that *FGFR* genomic aberrations are rare but recurrent, ranging from 0.3 to 5% in pan-cancer cohorts. However, genomic *FGFR* alterations are enriched in specific subtypes of difficult-to-treat paediatric brain tumours, sarcomas, and rare subtypes of haematological malignancies [10, 11, 16, 17]. Identifying and understanding the oncogenic function of these alterations and their sensitivity to targeted therapies is critical to improving the dismal outcomes currently facing paediatric patients with these cancers. Like *BCR-ABL* + ALL and *NTRK* fusion-driven cancers, the presence of true *FGFR* driver alterations has the potential to be targeted with an expanding range of novel FGFR inhibitors. Several have recently shown superior efficacy and lower toxicity over first-generation broad-spectrum FGFR-inhibitors in multiple cancer subtypes [18, 19]. It is thus an exciting and prescient time to assess the status of *FGFR* aberrations, their driver potential, and their targetability with clinically relevant drugs in paediatric cancers.

## Normal FGFR-pathway signalling: structure, function, and regulation of FGFRs

**Fig. 1:**
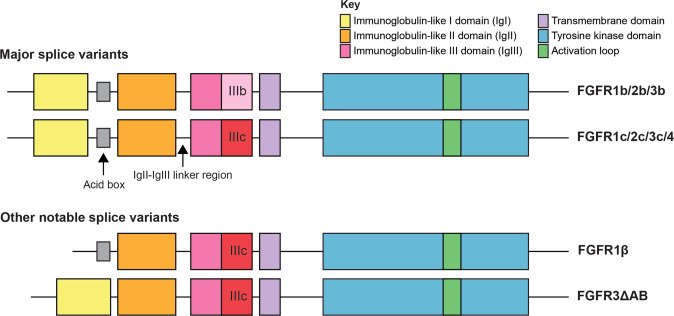
FGFR functional protein domains and splice isoforms. Schematic of FGFR functional domains and splice variants. Functional domains are coloured according to the key in the top right corner.

FGFR signalling functions in a range of normal physiological processes, including embryonic development, metabolism, and tissue homoeostasis. Studies in mice have demonstrated that *Fgfr1* and *Fgfr2* are required for normal development, and homozygous disruption of either of these genes results in embryonic lethality [20, 21]. Conversely, *Fgfr3*-deficient embryos are viable but have skeletal and inner ear defects [22], while disruption of *Fgfr4* alone has no overt effects on development [23]. The FGFRs have a conventional RTK structure consisting of an extracellular ligand binding domain, which binds fibroblast growth factor (FGF) ligands, a transmembrane domain, and intracellular kinase domain [1]. In humans, there are four FGFRs (FGFR1-4), which share high sequence homology of 55-72% [24], and 18 FGF ligands that interact with the FGFRs with varying binding affinities. FGFs have been reviewed in detail elsewhere [25], but they are broadly grouped into two classes, (1) canonical FGFs, containing five subfamilies of paracrine FGFs that require heparin/heparan sulphate as co-factors for receptor binding, and (2) endocrine FGFs, consisting of three FGFs that exhibit low heparin-binding affinity and can be released from the extracellular matrix and act as endocrine factors, requiring either αKlotho or βKlotho as co-factors [26]. Binding of these ligands to FGFRs is regulated by the immunoglobulin-like domains 1–3 (IgI, IgII, and IgIII) (Fig. 1). IgII and IgIII, and the linker region between these domains, are required for ligand binding and mediate specificity of FGF ligands and their receptors through alternative splicing of two exons (IIIb and IIIc) that encode the distal portion of the IgIII domain [27]. In addition to the alternative splicing of the IgIII domains of FGFR1-3, splice isoforms lacking the IgI domain (FGFR1 and FGFR2), the IgI-IgII linker region (FGFR3), or both (FGFR2) have also been described (reviewed in [28]). Notably, the IgIII domain of FGFR4 is not alternatively spliced [29]. Functionally, the IgI domain and the IgI-IgII linker region, containing an “acid box” consisting of eight acidic residues, are thought to inhibit ligand binding [24, 30]. In keeping with this, *FGFR1* transcript variants without the IgI-coding domain (termed FGFR1β) display increased FGF2 ligand binding affinity [31].

**Fig. 2:**
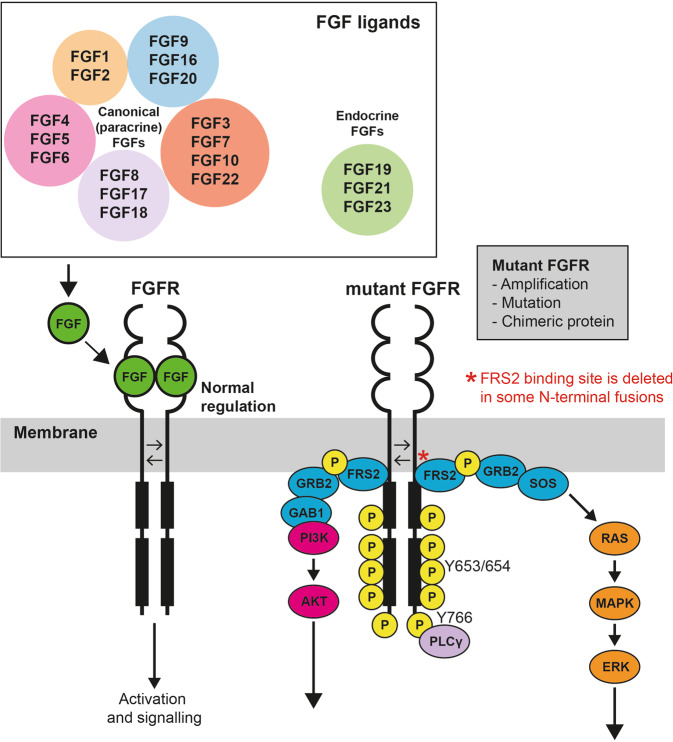
The FGFR pathway—regulation and downstream signalling. Overview of the normal regulation of FGFR kinase activation and consequences of mutant FGFR expression in cancer. Under normal conditions, binding of FGF ligands, including 18 canonical and three endocrine FGFs, mediates receptor dimerisation and transphosphorylation resulting in activation of the kinase. Mutation of FGFR, commonly the result of gene amplification, mutation or gene fusion, results in constitutive activation of the kinase domain and aberrant downstream signalling, leading to activation of multiple pathways, notably PI3K/AKT and RAS/MAPK.

Binding of FGF triggers FGFR dimerisation and autophosphorylation at specific tyrosine residues in the activation loop of the kinase domain, for example Y653 and Y654 in FGFR1 [32]. Activation of FGFR kinase leads to subsequent activation of several notable downstream cellular signalling pathways, including RAS/MAPK and PI3K/AKT (Fig. 2). There are two important mediators of this process, FGFR substrate 2 (FRS2), an adaptor protein that is constitutively associated with the FGFR and is phosphorylated upon kinase activation, and PLCγ, which binds to phosphorylated Y766 at the C-terminal of FGFR and activates protein kinase C (PKC) [33, 34].

## Abnormal FGFR signalling

Aberrant FGFR activation in cancer is commonly associated with one of several genomic events including gene amplification, mutation, and SVs, including gene fusions and other rare structural events such as internal tandem duplication (ITD). In addition, less well explored mechanisms of FGFR activation include *FGFR* alternative splicing and alternate transcript expression, and FGF ligand mediated activation. *FGFR* RNA overexpression in the absence of a genomic event remains unexplained in most instances, although there is some evidence to suggest that this might be a secondary phenomenon related to other oncogenic fusions [35]. The circumstances in which *FGFR* RNA expression is truly associated with FGFR activation remains an important conundrum to solve.

### Gene amplification

**Fig. 3:**
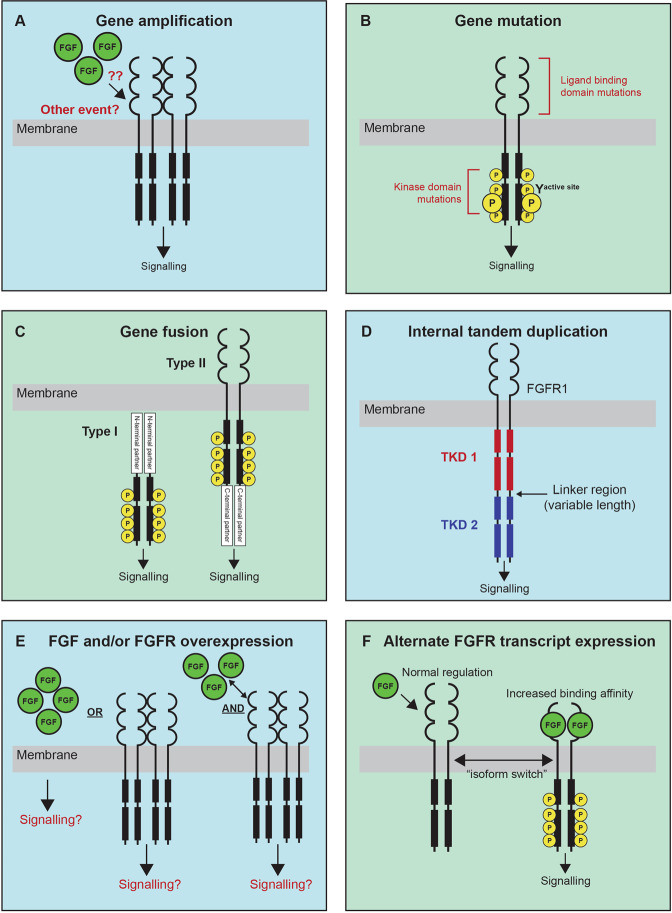
Mechanisms of FGFR kinase activation. **A** Gene amplification is predicted to result in an increase in FGFR protein expression and increased FGFR signalling. It is likely other events, including an increase in FGF ligand expression, cooperate with FGFR amplification to drive receptor activation. **B** Mutations in FGFRs most commonly occur in the ligand binding or kinase domain which result in increased ligand-binding affinity or ligand-independent receptor activation through a disruption of normal kinase autophosphorylation, respectively, leading to FGFR activation. **C** There are two classes of fusion genes which involve FGFRs, Type I –fusion of an N-terminal partner, or Type II—fusion of a C-terminal partner. Both N and C-terminal partners commonly contribute dimerisation domains that result in ligand-independent dimerisation and phosphorylation and activation of the FGFR kinase. **D** FGFR1 internal tandem duplication (ITD) results in a receptor with containing two tyrosine kinase domains (TKD1 and TKD2) separated by a linker region of variable length, which has been shown to promote kinase activation. **E** The functional result of increased FGF ligand or FGFR RNA or protein overexpression is currently unclear. It is possible that it is the combination of both ligand and receptor overexpression that will promote FGFR activation. **F** FGFR ligand binding domains are subject to splicing under normal conditions, but alternate isoforms lacking regions of the ligand binding domains have been shown to be upregulated in specific cancers. The common functional result of FGFRs that lack these domains is an increase in either ligand or heparin binding affinity which results in increased FGFR activation.

Gene amplification of *FGFRs* is thought to lead to increased protein expression and a functional dependency on FGFR signalling (Fig. 3A). The greatest functional evidence supporting this mechanism of activation comes from adult cancers, including *FGFR1*-amplified lung cancer and *FGFR1*- and *FGFR2*-amplified breast cancer [36, 37]. For example, *FGFR1*-amplified lung cancer cell lines exhibit increased FGFR1 protein expression compared to non-*FGFR1*-amplified cell lines and are sensitive to shRNA-mediated knockdown or pharmacological inhibition of FGFR1 [36]. Further, in breast cancer, *FGFR1*-amplified cell lines express higher levels of FGFR1 protein and are more sensitive to FGFR inhibition with the pan-kinase inhibitor, brivanib, compared to cell lines with normal *FGFR1* or *FGFR1* deletion [37]. Despite these observations, it is notable that ectopic overexpression of wild-type (WT) *FGFR1*, which in essence recapitulates copy number gain, is not sufficient to transform cytokine dependent cell lines (Ba/F3) and requires addition of exogenous FGF2 (also known as bFGF) ligand and heparin to stimulate proliferation [38]. This contrasts with FGFR1-activating mutations or fusion genes that transform these same cytokine-dependent cell lines [39, 40]. It is likely then, that cooperation between *FGFR* amplification and ligand overexpression, or another co-operating activating mechanism, results in FGFR activation. This highlights the importance of interrogating the genomic and transcriptomic context of *FGFR* amplification to determine if specific FGF ligands might be driving FGFR signalling.

**Fig. 4:**
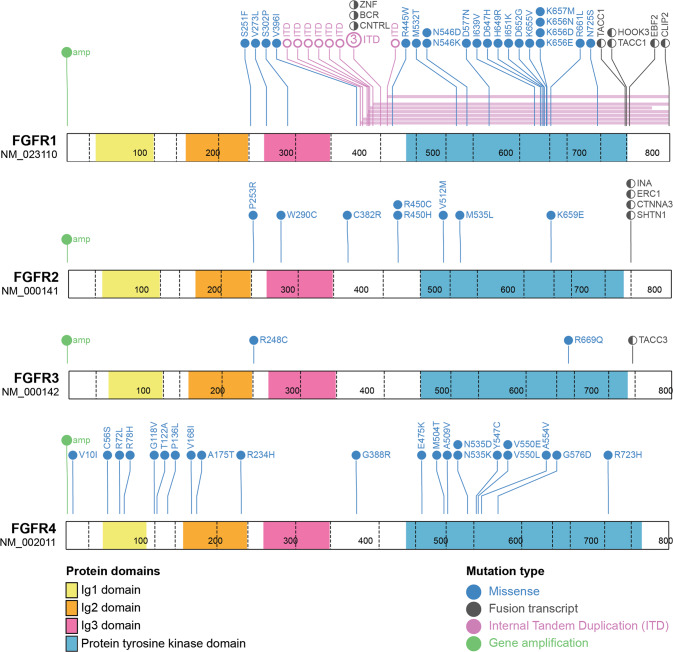
FGFR variants in paediatric cancers. Schematic of protein outcomes of *FGFR* genomic and transcriptomic aberrations in paediatric cancer. Functional protein domains and mutation types are coloured according to the key. Single cases of *HOOK3-FGFR1* and *FOXO1-FGFR1* gene fusions have also been reported in paediatric cancers but the molecular structure of these fusions is either unknown or unreported and as such are not included in this figure. Figures were generated using Protein Paint [128]. A list of genomic variants used to generate this figure, and the specific paediatric cancer subtypes they have been identified in is included as Supplementary Table 1.

In paediatric cancers, *FGFR* amplification is rare but predominately identified in sarcomas, particularly osteosarcoma (OS), and less frequently in brain tumours, with gain of *FGFR1* being most common (Fig. 4 and Supplementary Table 1). In the Ma et al and Grobner et al pan-cancer genomic studies [10, 11], comprising 2670 paediatric tumour samples of 24 cancer types combined, *FGFR* amplification was identified in only two cases (0.075%), both involving *FGFR1*, in one rhabdomyosarcoma (RMS) and one OS sample. When studies selecting for high-risk patients (relapsed, recurrent/refractory, or high-risk subtype diagnosis) are analysed alone: MOSCATO-01 [14]; the Zero Childhood Cancer Program (ZERO) [12]; the Paediatric Precision Oncology INFORM Registry [15]; the MAPPYACTS Trial [16], the NCI-COG Paediatric MATCH Trial [41], and the GAIN/iCAT Study [42], *FGFR* amplification is identified at a higher frequency, in 28 of a combined 3451 samples (0.81%). Recent interrogation of two paediatric cancer cohorts (The Dana-Faber/Boston Children’s Profile Study and the GAIN/iCAT2 study) comprising a combined total of 1395 patients, identified FGFR amplification in 11 tumours (0.8%). This included *FGFR1* amplification in RMS (*n* = 3), Ewing sarcoma (ES; *n* = 2), OS (*n* = 1) and a sarcoma other (*n* = 1); *FGFR3* amplification in a CNS tumour (*n* = 1); and *FGFR4* amplification in RMS (*n* = 3). In a cohort unselected by age of 275 osteosarcomas arising in the bone (age range: 4–64 years), *FGFR1* amplification was detected in 24 (~10%) patients, including paediatric patients, and was disproportionately present in the rarer histological variants and associated with poor response to chemotherapy [43]. While pre-clinical data is limited, one study demonstrated that *FGFR1* amplification in 1/17 primary osteosarcoma samples predicted sensitivity to the selective FGFR-inhibitor NVP-BGJ398 [44]. Notably, *FGFR* amplification is almost never identified in paediatric haematological maliganancies, including the most common paediatric tumour type, B-ALL.

### Gene mutation

Gene mutations, or SNVs, in *FGFR*s typically occur in the ligand binding or kinase domains, and functionally result in increased ligand binding affinity or ligand independent receptor activation (Fig. 3B) [45]. The FGFR1 N546K kinase domain mutation has been shown to disrupt the sequential three-step autophosphorylation of normal FGFR1[32], inducing phosphorylation at the active site, Y653, at a rate 25 times that of WT FGFR1 [46]. This change in autophosphorylation has also been observed for the FGFR3 K650E mutation, which promotes an overall increase in phosphorylation on the activation loop residues (Y647 and Y648) and broad phosphorylation of additional sites compared to WT FGFR3 [47]. Functionally, mutations in the active site, including the FGFR3 K650E mutation, and its analogous mutations in FGFR1 (K656E) and FGFR4 (K645E), induce FGFR activation and transformation of NIH3T3 cells [48]. FGFR4 N546K and V550E mutants have been shown to increase FGFR4 autophosphorylation, STAT3 signalling, proliferation, and metastatic potential when expressed in a murine RMS cell line [49]. A recent high-throughput functional analysis of *FGFR* aberrations (largely gene mutations and a small number of fusions) assessed the transforming activity of 158 FGFR mutations, in comparison to the respective WT FGFR. Importantly, of 122 variants of unknown significance (VUS) tested, 25 were determined to be oncogenic or likely oncogenic (Table 1), highlighting the breadth of functionally important mutations in these genes [50].

**Table 1:** FGFR VUS variants characterised as oncogenic or likely oncogenic using an in vitro high-throughput transformation assay (MANO method).

	Likely oncogenic	Oncogenic
FGFR1	N546K; K656E	K656M
FGFR2	C62Y; N82K; E160K; E163K; Q212K; A264T; K310R; R399X; H416R; I422V; H544Q; L560F; S791T	A67V; D101Y; Y328N; G364E; V392A; E718K
FGFR3	H284fs*10	R248 S249insC; K650Q

Most *FGFR1/2/3* SNVs are sensitive to second generation FGFR-selective inhibitors (for example, AZD4547 and erdafitinib) that reversibly bind to the ATP-binding pocket of the kinase and thus prevent phosphorylation at the active site. However, mutations in the active site, including FGFR1 N546K and FGFR2 N549D/K, confer resistance to most FGFR-selective inhibitors, with the exception of moderate responses observed towards irreversible FGFR inhibitors (E7090 and futibatinib), which covalently bind to the kinase and cannot be displaced by ATP [50]. In the context of gatekeeper mutations, including FGFR1 V561M, resistance is most commonly attributed to steric hinderance, caused by the replacement of the gatekeeper residue with a hydrophobic amino acid that stabilises the active conformation and reduces affinity to inhibitors [51]. However, analysis of the interaction between FGFR1 V561M and various ATP-competitive inhibitors has demonstrated that inhibitors with binding flexibility can still bind to this active conformation (AZD4547), while for others the mechanism of resistance remains unexplained (ponatinib and dovitinib) [51, 52]. Functional analysis of the FGRF1 N546K specifically has demonstrated that this mutation does not affect the affinity of the mutant for binding ATP-competitive inhibitors (ponatinib, dovitinib, PD173074 and BGJ-398), but instead increases affinity of FGFR1 to ATP [52]. However, as with the gatekeeper mutation, it is likely that the mechanism of resistance is also dependent on the structure of the inhibitor. Indeed, it has been suggested that FGFR1-activating mutations may also prevent binding of specific inhibitors, as they are fixed in the active “closed” conformation and are often complexed with ATP analogues, while FGFRs are commonly in “open” conformation when complexed to ATP-competitive inhibitors [50]. Due to the structural differences between FGFR4 and the other FGFRs, targeting FGFR4 SNVs, including the V550L gatekeeper mutation, with pan-FGFR inhibitors is largely ineffective [50]. However, RMS cell lines expressing these mutants have been shown to be more sensitive to the FGFR-selective inhibitor PD173074 and pan-kinase inhibitor ponatinib, compared to cell lines expressing WT FGFR4 [49, 53].

Mutations in *FGFR* genes have been identified in a wide range of paediatric cancer types. These include established activating FGFR mutations, FGFR1 N546K and K656E in low-grade (LGG) and high-grade glioma (HGG), neuroblastoma, medulloblastoma, glioblastoma, and Wilms tumour, and FGFR4 N535K/D and V550L/E in RMS [11, 12, 41, 42, 49, 54–57]. Whole genome sequencing of 149 paediatric LGG patients demonstrated that *FGFR* aberrations are recurrent in LGG but predominately consist of SVs (including gene fusions and ITDs), only reporting the FGFR1 N546K mutation in one patient [54]. Molecular profiling of 91 uncommon cerebral low-grade neuroepithelial tumours (LGNTs), identified *FGFR1*, *FGFR2*, and *FGFR3* aberrations in 30 (33%), two (2%), and one (1%) patient/s, respectively [58]. The authors demonstrated that *FGFR1* aberrations were characteristic of LGNTs with an oligodendroglial phenotype (dysembryoplastic neuroepithelial tumours (DNETs) and oligodendrogliomas), identified in 62% of patients, but were a less common feature of astrocytic tumours and gangliomas. Of note, the authors described four cases of two *FGFR1* mutations occurring on the same allele and one case of *FGFR1* mutations occurring on different alleles in single patients. Further, the authors also identified co-occurrence of *FGFR1* mutations with other driver mutations (*BRAF* or *NF1*) in two patients suggesting that while this does occur its relatively rare (2/30 patients). A more recent study of over 1000 paediatric LGGs identified *FGFR1* hotspot mutations, primarily consisting of N546K and K656E, in 1.5% of their cohort, suggesting that they may be more frequent in LGG [59]. *FGFR* aberrations are incredibly rare in HGG, and include a handful of cases of FGFR1 N546K [12, 56], FGFR1 K656E [11], FGFR1 N546D [14], and FGFR1 D577N [14]. Analysis of a cohort of 94 paediatric RMS patients identified 14 *FGFR4* missense mutations, including six mutations in the tyrosine kinase domain (TKD) (N353D/K, V550E/L, A554V, and G576D), which were more commonly identified in *PAX3-FOXO1* fusion negative RMS [49]. FGFR SNVs are not commonly associated with other paediatric sarcoma subtypes and are only rarely identified in haematological malignancies, including in B-ALL where *FGFR* activating mutations are identified in <1% of cases [10, 60].

### Gene fusions and other structural variants

Gene fusions involving *FGFRs* have been identified in a range of paediatric and adult cancers. There are two classes of fusion genes which involve either an N-terminal partner (termed type I) or a C-terminal partner (termed type II). In either case, the fusion partners commonly contribute protein-protein interaction domains, facilitating the dimerisation and activation of FGFR kinase signalling [45] (Fig. 3C). Type I fusions generally involve the loss of the N-terminal ligand binding (regulatory) and transmembrane (membrane localisation) domains of FGFRs and are commonly identified in haematological malignancies. Single cases of FGFR1-activating fusions have been reported in B-ALL (*HOOK3-FGFR1)* and RMS *(FOXO1-FGFR1)*, although the exact molecular structure of these fusions is unknown [61, 62]. Type I FGFR1 fusions are associated with “myeloid/lymphoid neoplasms with *FGFR1* rearrangement”, an extremely rare myeloproliferative disorder characterised by the presence of *FGFR1* rearrangement. This disease primarily affects adults but has been described in 16 childhood or adolescent (≤21 years of age) cases in the literature [17, 40]. In this age group, the disease primarily presents as eosinophilia and T-cell lymphoblastic lymphoma with a short chronic phase that can transform to acute myeloid leukaemia [17]. While at least 11 *FGFR1* partner genes have been reported in adult cases, only three have been reported in paediatric cases, *CNTRL* (formerly *CEP110*), *ZMYM2* (formerly *ZNF198*) and *BCR* [17, 40].

Functional analysis of the BCR-FGFR1, CNTRL-FGFR1, and ZNF198-FGFR1 fusion proteins in Ba/F3 cells has shown that each of these fusions can transform cells to cytokine independence and are sensitive to inhibition with FGFR-targeting inhibitors, including ponatinib and dovitinib [39, 40, 63, 64]. Further analysis of BCR-FGFR1 and ZNF198-FGFR1 has demonstrated the mechanism of activation mediated by BCR or ZNF198 and downstream signalling activation differs between these fusions. ZNF198-FGFR1 is localised to the cytoplasm, forms a dimeric structure, activates STAT proteins in a similar manner to WT FGFR1, and induces relatively increased phosphorylation and activation of STAT1, STAT3 and ERK1/2 compared to BCR-FGFR1. [65]. Importantly, in addition to autophosphorylation of key tyrosine residues in the activation loop of FGFR1, the same as ligand stimulated FGFR1, the ZNF198 region of the fusion protein itself has been shown to be phosphorylated at seven tyrosine residues that could act as docking sites for signalling proteins [66]. The development of a kinase-dead BCR-FGFR1 construct, by introducing the K514A mutation in the FGFR1 region, demonstrated that FGFR1 kinase activity is essential for STAT and MAPK activation, and cellular transformation [67]. Further, mutation of phosphorylation sites within the BCR region of the fusion, excluding the GRB2 binding site Y177, had little effect on the transforming capacity, but mutation of key residues within the coiled-coil domain, responsible for salt-bridge formation, abolished the transforming capacity. Taken together, these data demonstrate that although FGFR1 kinase activation is the primary transforming event for FGFR1 fusions, fusion partners likely mediate different mechanisms of activation and intermolecular interactions.

There is currently no consensus for the clinical management of paediatric myeloid/lymphoid neoplasms with an FGFR rearrangement and treatments vary based on symptoms and haematological condition at diagnosis [17]. Of available data, 42% patients (5/12) succumb to their disease (follow-up time varies from 1.4-13 years) suggesting that new therapies are urgently needed [17, 40]. Data supporting the potential efficacy of FGFR-targeted TKIs in this disease is mostly limited to pre-clinical studies [40, 63, 64]. The potential clinical efficacy of ponatinib has been demonstrated in an adult patient with *BCR-FGFR1* + trilineage mixed-phenotype acute leukaemia [68], but there is very limited data available in paediatric patients [40]. Whether FGFR1-targeting TKIs could improve outcomes for patients with this disease is yet to be established.

Type II fusions generally retain most functional domains of FGFRs including the ligand binding, transmembrane domain and TKD, and are more commonly identified in solid tumours. Functionally, these fusions are membrane bound and constitutively activated by the addition of C-terminal dimerisation domains. In paediatrics, Type II fusions are most frequently associated with various subtypes of LGG [42, 55, 59], most notably the *FGFR1-TACC1* and *FGFR3-TACC3* fusions, but have also been identified in rare cases of other tumour types including *FGFR1-ERC1* in HGG [12], *FGFR1-HOOK3* in a young adult gastrointestinal stromal tumour (GIST) [69], and *FGFR1-EBF1* in spindle cell sarcoma [42] (Fig. 4). *FGFR2* or *FGFR3* gene fusions are also a characteristic molecular feature of a recently described subtype of low grade neuroepithelial tumour—Polymorphous Low-grade Neuroepithelial Tumour of the Young (PLNTY) [70]. Interestingly, gene rearrangements including *FGFR1* ITD, *FGFR1* fusions, and *FGFR2* fusions, have been classified as low risk in LGG, compared to *FGFR1* mutations which are associated with intermediate risk and inferior overall survival [34].

The *FGFR1-TACC1* and *FGFR3-TACC3* fusions transform rat fibroblasts and induce glial-like tumour formation when ectopically expressed in immunodeficient mice [71]. When expressed in astrocytes, the FGFR3-TACC3 fusion is localised to spindle microtubules and may not activate canonical FGFR downstream signalling pathways, PI3K/AKT or MAPK. Interestingly, FGFR3-TACC3 and FGFR1-TACC1 expression induces a range of mitotic errors, suggesting in this context it is largely the TACC region of the protein that is contributing to the function of the fusion. Despite this, FGFR inhibitors are effective at killing FGFR-TACC-expressing cells in vitro and prolong survival of mice bearing FGFR3-TACC3-induced tumours in vivo, suggesting the TK activity is important. Interestingly, mutation of tyrosine residues that are normally phosphorylated in the TACC3 region of the fusion increased colony formation in NIH3T3 cells and IL-3 independent proliferation in 32D cells, suggesting that phosphorylation of these residues may have a negative regulatory effect on signal transduction [47]. These data suggest that FGFR-TACC fusions may be weaker oncogenic drivers and may provide a mechanistic explanation for the favourable outcomes observed in LGG patients with FGFR fusions, compared to FGFR mutations [59].

In addition to gene fusions, other SVs, including *FGFR1* ITD which results in a duplication of the TKD at a protein level (Fig. 3D), also promote FGFR1 kinase activation [54]. FGFR1 ITD is the most frequently observed *FGFR* aberration in paediatric LGG [54, 59] but there are few studies that have investigated the exact mechanism of kinase activation. FGFR1 ITD results in an FGFR1 protein with two TKDs (TKD-1 and TKD-2) separated by a linker region of variable length (74-104 amino acids) [54]. Transplantation of *Tp-53* null astrocytes transfected with FGFR1 ITD into the brains of nude mice rapidly induces astrocytic tumour formation, characterised by PI3K/AKT and MAPK pathway activation, demonstrating that this alteration is sufficient for oncogenesis. Further, the FGFR inhibitor, PD173074 was shown to effectively block autophosphorylation of FGFR1 ITD, suggesting that these inhibitors may be clinically effective in patients with FGFR1 ITD. Much like FGFR gene fusions, FGFR1 ITD positive tumours are thought to be less aggressive than FGFR mutated tumours [59]. Of note, a single case of FGFR1 ITD has also been reported in neuroblastoma where the duplicated region spans part of exon 17 encoding the kinase domain [57]. Whether this would functionally result in FGFR1 activation was not investigated, which highlights the value of model systems to functionally validate the novel molecular findings that will inevitably come with sequencing more patients.

### Other mechanisms: transcriptional upregulation of FGFRs and FGF ligand overexpression

The application of transcriptome sequencing in pan-cancer studies has highlighted the frequency and potential importance of increased RNA expression of RTKs, including FGFRs, and their ligands. Here, we distinguish increased RNA expression arising in the absence of an associated gene amplification or mutational event. In such instances, increased FGFR RNA expression may arise as a result of mutations in non-coding or regulatory regions, epigenetic dysregulation, or as a secondary consequence of other oncogenic drivers [45]. The principal question is under what circumstances is overexpression of a WT FGFR indicative of FGFR signalling activation and, more pertinently from a therapeutic standpoint, when is it indicative of tumour dependence on FGFR signalling? While this question is still open, it is likely that a combination of specific factors, including FGF ligand overexpression, are associated with receptor activation (Fig. 3E). Some of this evidence comes from studies of *FGFR* gene amplification, the consequence of what is essentially WT FGFR expression. Overexpression of FGF ligands has been identified in multiple cancer types and is recurrently associated with aggressive tumour types and inferior clinical outcomes. Studies exploring the role of FGF ligands in cancer are mostly limited to adult cancer subtypes and include FGF2, the most commonly implicated FGF in cancer, FGF1 in ovarian, FGF4, FGF9 and FGF22 in lung, FGF5 in breast, FGF6, FGF19, and FGF21 in liver, FGF7 and FGF18 in gastric, and FGF8, FGF17, FGF19, and FGF23 in prostate cancer (reviewed in [72]). Most of these studies have focussed on the prognostic implications of increased FGF expression but have not explored the functional implications on FGFR signalling activation or corresponding therapeutic sensitivity.

There are few studies that have established clear obligate relationships between specific FGFRs and FGFs in the context of cancer development and progression, and these again are mainly limited to adult cancers. For example, FGF19, which is commonly amplified in hepatocellular carcinoma, promotes hepatocyte proliferation through activation of FGFR4 [73]. In addition, FGF7 and FGF18 are thought to cooperate with FGFR2 amplification to promote gastric cancer [74, 75]. In non-small cell lung cancer (NSCLC), FGF/FGFR pathway activation, mediated by FGF2 and FGF9 signalling through FGFR1 or FGFR2, has been suggested to promote EGFR inhibitor resistance [76].

Recently, paediatric precision medicine trials have started to include the analysis and reporting of RNA overexpression of targetable genes and have highlighted *FGFR* overexpression as a feature of multiple subtypes of paediatric cancer [12, 15]. The ZERO program was the first paediatric precision medicine program to publish reportable findings of RNA overexpression, using a z-score and fold change cut-off of greater than 2 to determine outlier overexpression, when compared to the entire cohort (all tumour types). In the first 252 tumours published from ZERO, overexpression of *FGFR* genes was reported in 16 tumours (6.3%), dominated by *FGFR4* overexpression in fusion positive RMS (9 tumours), in which *FGFR4* overexpression is a known molecular feature and is directly regulated by PAX3-FOXO1 [77]. The INFORM study reported RNA outlier expression, in the absence of genomic aberration, of either *FGFR* (most commonly *FGFR3* or *FGFR4*) or *FGF* genes in 32 and 8 cases, respectively, from a total of 781 patients with RNAseq data [15]. Of note, combined overexpression of both *FGFR* and *FGF* genes was not identified in any of the tumours analysed. In both ZERO and INFORM, aberrant expression of FGFR pathway genes was restricted to sarcomas, brain and CNS tumours, and solid tumours. This observation, together with the low reported frequencies of genomic aberration of FGFR pathway genes, suggests that the FGFR pathway may be less important in haematological malignancies.

*FGFR1*, *FGFR4* and *FGF8* expression is recurrently altered in RMS. The prevalence of *FGFR1* overexpression in the two histological subtypes of RMS, alveolar RMS (aRMS) and embryonal RMS (eRMS) has varied in different patient cohorts, with some studies reporting higher levels of expression in eRMS [78], while others have identified higher levels in aRMS but only in *PAX3-FOXO1* negative cases [79]. One study identified *FGFR1* overexpression in the absence of genomic alteration in both RMS primary samples and cell lines and showed that it was associated with hypomethylation of a 5‘ CPG island and abnormal expression of *AKT1*, *NOG* and *BMP4* genes in primary samples [80]. *FGFR4* overexpression in RMS is associated with the aRMS histological subtype, which is molecularly characterised by the *PAX3/7-FOXO1* fusion, advanced-stage disease, and poor survival [49]. While FGFR4 is an established transcriptional target of PAX3-FOXO1 in fusion positive RMS, and increased *FGFR4* RNA and protein expression is frequently identified in fusion positive RMS patients, the dependency on and as such therapeutic targetability of FGFR4 in this disease is debated. The oncogenic potential of WT FGFR4 is supported by the findings that FGFR4 knockdown reduced tumour growth in a human RMS cell line and lung metastases in a RMS mouse model [49], and WT FGFR4 overexpressing mouse-myoblasts induced tumour formation in vivo [81]. However, the transforming potential of WT FGFR4 was not as aggressive as FGFR4-activating mutations, and most WT FGFR4 tumours lacked RMS-specific immunohistochemistry markers [81]. Importantly, cells expressing WT FGFR4 are substantially less sensitive to FGFR inhibitors, such as ponatinib, compared to cells expressing FGFR4-activating mutants, N535K and V550E [49, 53]. Based on these preclinical studies, targeting overexpressed WT FGFR4 is currently considered to be only minimally effective in RMS. Recently, FGF8 was identified as a direct transcriptional target of PAX3-FOXO1, where FGF8 upregulation represents a novel mechanism of PAX3-FOXO1 independent growth in aRMS [82]. More research is needed to determine whether specific targeting of FGF8 in *PAX-FOXO1*-positive RMS is an effective treatment strategy.

In addition to RMS, aberrant expression of FGFR pathway genes has also been identified in OS, Ewing sarcoma (ES), Malignant Peripheral Nerve Sheath Tumour (MPNST), and synovial sarcoma (SS). There are a multiple studies in OS cell lines that have used either phosphoproteomics or immunohistochemistry methods to investigate the expression and activation of FGFRs in small numbers of OS cell lines, where conclusions have been mixed but FGFR1, FGFR2, FGFR3, and FGF2 have all been implicated [83–85]. Notably, one study suggested that APE1 promotes angiogenesis in OS through upregulation of FGFR3 and FGF2 [85]. A preclinical study using in vivo transgenic models of c-Fos oncogene-induced OS identified FGFR1 as a novel c-Fos/AP-1 regulated gene. Expression of c-Fos induced an increase in FGFR1 RNA and protein, promoted anchorage-independent growth, and MAPK activation in a FGF2-dependent manner. Pharmacological inhibition of FGFR1 blocked colony forming of OS cells in vitro and reduced OS metastasis in vivo [86]. In ES, one study showed that FGFR1 was expressed and activated in 78% of primary ES cells and FGF2 stimulation induced motility and invasion of these cells [87]. In addition, another study identified *FGFR4* as a potential gene dependency in ES using an siRNA screen targeting 287 cancer associated genes in four ES cell lines. Further, subsequent treatment with the selective FGFR4 inhibitor BLU9931 inhibited ES cell growth [88]. In MPNST, combined expression of FGFR1 and FGFR2 is associated with superior overall survival, while FGFR4 expression is associated with poor disease-free survival [89]. FGFR3 has also been postulated to be a positive prognostic marker in SS, with high FGFR3 being associated with improved progression free survival in untreated SS patients [90]. Expression of the *SS18-SSX2* fusion, which molecularly characterises SS, results in transcriptional upregulation of *FGFR2* and increased expression of FGF ligands, *FGF3*, *FGF7*, *FGF9* and *FGF18*, suggesting possible autocrine feedback loops function to upregulate FGFR signalling [91]. Another study showed that either *Fgfr1*, *Fgfr2*, or *Fgfr3* gene knockout impeded *SS18-SSX2*-induced tumour formation in vivo and FGFR inhibitor (BGJ398) treatment reduced SS tumour cell growth in vitro and in vivo [92]. Analysis of *FGFR* and *FGF* mRNA expression in SS primary tumours and cell lines demonstrated that these lines express most *FGFR*s and some *FGF* ligands, including FGF2, FGF8, FGF9, FGF11 and FGF18. Further, FGF8 stimulation of SS cell lines promoted proliferation and FGFR inhibitor treatment demonstrated efficacy in in vitro and in vivo, via downregulation of ERK [93]. These data together demonstrate that the FGFR pathway may play a role in multiple sarcoma subtypes.

In paediatric brain tumours, elevated expression of FGFRs have been reported in medulloblastoma and ependymoma, two common paediatric brain tumours in which genomic aberration of FGFRs is not a characteristic [11, 94]. Increased *FGFR3* expression has been associated with treatment failure in medulloblastoma [95], and FGFR inhibitor treatment has demonstrated some efficacy, albeit at high doses, in some medulloblastoma cell lines [96]. In ependymoma, high *FGFR1* and *FGFR3* expression has been associated with high tumour grade and poor prognosis, in the absence of *FGFR1* or *FGFR3* mutation [97]. Co-expression of FGFR1/3 with FGFs including FGF1, FGF2 and FGF9 has been identified in specific molecular subtypes and cell sub-populations of ependymoma. FGF2 has also been implicated in glioblastoma multiform (GBM), a grade IV HGG, which also frequently overexpresses *FGFR1* [98].

### Other mechanisms: alternative splicing of FGFR RNAs

As described earlier, alternative splicing of the FGFR Ig domains occurs normally and mediates ligand binding specificity. This aspect of normal FGFR regulation has also been implicated in cancer. Alternative splicing of the distal portion of the IgIII domain in *FGFR1-3* results in the inclusion of either IIIb or IIIc exon and expression of these isoforms for FGFR1 and FGFR2 is generally restricted to epithelial cells for FGFR IIIb and mesenchymal cells for FGFR IIIc (reviewed in [25]). Further, these isoforms generally bind FGF ligands that are expressed in the reciprocal tissue, and this plays a key role in organ development. This pattern of expression and signalling by FGFR3 isoforms is not strictly observed. The majority of FGF ligands preferentially signal through FGFR IIIc isoforms, except for the FGF7 subfamily (FGF3, FGF7, FGF10, and FGF22) that signals through FGFR IIIb isoforms [25, 99]. In the context of cancer, aberrant signalling of FGF10 through FGFR2 IIIb, and to a lesser extent FGFR1 IIIb, has been implicated in a range of cancers [100]. For example, in breast cancer, FGF10 promotes cell migration through activation of FGFR1 IIIb, promoting translocation to the nucleus and transcription upregulation of cell migration genes, and activation of FGFR2 IIIb, promoting PI3K/SH3BP4 recruitment and receptor recycling [101, 102]. In paediatric cancer subtypes, there are few studies that have investigated the role of alternate splicing of FGFR IgIII domains. However, one study has shown that FGFR1 and FGFR3 IIIc splice variants are the dominant expressed isoforms in ependymoma [103]. In addition, expression of the FGFR1 IIIc isoform and FGF5 is elevated in astrocytoma and GBM patient samples compared to non-malignant controls, and FGF5 promotes oncogenic cell growth and survival through autocrine and paracrine signalling through the FGF/FGFR signalling axis [104].

Splice isoforms lacking the IgI domain and/or the IgI-IgII linker region of FGFRs also occur naturally (Fig. 3F), the most important of which is FGFR1β, which lacks the IgI domain and is upregulated in a range of cancers, including astrocytoma, pancreatic, breast, and bladder cancer [105–108]. Further, an increase in the ratio of FGFR1β to the full-length isoform (FGFR1α), is associated with more aggressive tumours and inferior clinical outcomes in the tumours in which it has been described. Recently, *FGFR1* was shown to be preferentially spliced, resulting in the FGFR1β isoform, in paediatric HGG compared to normal brain, which was also accompanied by upregulation of total *FGFR1* expression [109]. Functionally, FGFR1 isoform switching alters affinity for particular FGF ligands in bladder cancer cell lines [108]. In addition to FGFR1β, other splice isoforms, including FGFR3ΔΑΒ, an FGFR3 isoform that lacks the IgI-IgII linker region, containing the acid box (AB), have been shown to alter heparin binding and FGF affinity and represent another mechanism of FGFR activation that may be exploited in cancer [110].

## Co-occurring mutations in FGFR-driven paediatric cancers

The presence of co-occurring mutations, and potential co-operation, with FGFR alterations in paediatric cancer is not completely understood, largely due to the rarity of these mutations across the spectrum of paediatric cancer. Recently, specific analysis of *FGFR* aberrations in paediatric cancer by Lazo De La Vega and colleagues has characterised the breadth of co-occurring mutations in FGFR-driven paediatric maliganancies. In the 41 cases in which an activating FGFR alteration was identified, 66% of cases (27/41) had at least one additional oncogenic or likely oncogenic mutation in another gene [42]. Amplification of *MYC/MYCN* (7 cases) and mutation of *TP53* (6 cases) were the most common co-occurring mutations observed, as well as smaller numbers of cases of *MDM2/4* amplification and *CDKN2A/2B* deletion. Importantly, mutations in therapeutically targetable genes were also observed including *PIK3CA* mutations (3 cases), *CDK4* amplification (4 cases), and mutations in the RAS pathway (*NF1/2*, *NRAS*, *KRAS*, *HRAS*; 7 cases), highlighting potential opportunities for combination therapy approaches. Indeed, initial analysis of the molecular profiles of the first 1000 patients enroled in the NCI-Paediatric MATCH trial also identified co-occurrence of *PIK3CA* mutation (1 case), CDK4/6 amplification (3 cases), and RAS pathway activating mutations (*NRAS*, *PTPN11*; 4 cases) in 40 patients identified with *FGFR* alterations [41]. Notably, in both studies, co-occurring mutations did not appear to be associated with specific FGFR alterations or tumour types, although larger cohorts would be needed to determine any association. In LGG, FGFR alterations have been observed in patients with germline *NF1* mutations, and in combination with BRAF V600E, *CDKN2A* deletion, and mutations in *PIK3CA*, *NRAS*, *MAPK2*, *SET2D*, and *JAK2* [59]. In addition, dual mutations in *FGFR1* are also observed. Whether these mutations co-operate to drive tumorigenesis, or they represent potential combination therapy options, is yet to be explored.

## Recent developments in FGFR targeted therapies

In recent years, a range of FGFR-inhibitors have been developed and tested in pre-clinical and, in some instances, clinical studies. First generation multi-target TKIs (eg. ponatinib, dovitinib and lenvatinib) inhibit a range of TKs, including but not limited to VEGF1/3, KIT, and RET, in addition to FGFRs. The broad-spectrum activity of these inhibitors has resulted in a lack of anti-FGFR activity and the occurrence of adverse events, demonstrated in a Phase II clinical trial of dovitinib for FGFR2-mutated metastatic endometrial cancer [111]. A number of these broad-spectrum multi-kinase inhibitors have been trialled clinically, without molecular indication, in a range of sarcoma subtypes where efficacy was mainly observed in OS. Regorafenib and sorafenib have been shown to modestly prolong progression-free survival (PFS) of OS patients [112–114], and regorafenib has demonstrated comparable efficacy in ES patients [115, 116] (Table 2). Lenvatinib, which has a similar target profile, demonstrated similar clinical efficacy to regorafenib when used as a monotherapy and tested specifically in a young patient cohort (2-25 years) with relapsed/refractory OS, where disease control and objective responses of 48% and 6.7%, respectively, were achieved [117]. However, when combined with etoposide and ifosfamide, disease control and objective response rates of 71% and 9%, respectively, were achieved [118]. A partial response to ponatinib has also been reported in a paediatric oligodendroglioma patient with an *FGFR3-PHGDH* fusion [119]. As with any multi-kinase inhibitor, it is however very difficult to assess how much of this anti-tumour effect, if any, can be attributed to inhibition of the FGFR-pathway. Although with these inhibitors the overall toxicity was manageable, for some patients these can be severe. It should be noted that the efficacy of Regorafenib will also be tested in combination with immunotherapy in OS (NCT04803877), and with chemotherapy in relapsed RMS (FaR-RMS, NCT04625907), without molecular indication, in clinical trials. In addition, surufatinib will be tested in paediatric patients with relapsed or refractory solid tumours with *FGFR1* aberrations (NCT05093322) (Table 2).

**Table 2:** FGFR targeted therapies in clinical trial and case studies in paediatric and young adult cancer.

Drug	Targets	Trial	Tumour types	Response rate	Refs
*Multi-kinase inhibitors*					
**Regorafenib**	**FGFR1**, RET, VEGFR, KIT, PDGFR	SARCO24 (Phase II, NCT02048371)	Advanced liposarcoma, osteogenic sarcoma, rhabdomyosarcoma, and Ewing family sarcoma (≥10 years of age)	**Osteosarcoma:** Median PFS 3.6 months (vs. 1.7 months control) **Ewing sarcoma:** Median PFS 3.4 months	[112], [116]
		REGOBONE (Phase II, NCT02389244)	Metastatic bone sarcomas: conventional high-grade osteosarcoma, Ewing sarcoma of bone, intermediate or high-grade chondrosarcomas and chordomas and either bone or soft tissue metastatic CIC-rearranged sarcomas (≥10 years of age)	**Osteosarcoma:** 8-week non-progressive disease 65% (17/26 patients); 2 PR and 15 SD Median PFS 3.8 months (vs. 0.9 months control) **Ewing sarcoma:** 8-week non-progressive disease 57% (13/23 patients); 5 PR and 8 SD Median PFS 2.6 months (vs. 0.9 months control)	[113], [115]
		FaR-RMS (Phase I/II, NCT04625907)	Children and adolescents with newly diagnosed rhabdomyosarcoma	Recruiting, response data yet to be reported	NA
		SARC038 (Phase II, NCT04803877)	Combination of regorafenib and with nivolumab in patients with refractory or recurrent osteosarcoma (≥5 years of age)	Active, not recruiting	NA
**Sorafenib**	**FGFR1**, RAF, VEGFR, KIT, PDGFR, FLT3	Phase II (NCT00889057)	Relapsed and unresectable high-grade osteosarcoma (≥14 years of age)	4-month PFS 46% (*n* = 35); median PFS 4-months; OS 7-months; 3 PR, 2 MR, 12 SD	[114]
**Lenvatinib**	**FGFR1/2/3/4**, RET, VEGFR, PDGFR, KIT	Phase II (NCT02432274)	Relapsed/ refractory osteosarcoma (aged 2-25 years)	**Monotherapy:** 4-month PFS 29%; median PFS 3-months; ORR 6.7%; 2 PR and 13 SD **Lenvatinib** + **etoposide/ifosfamide:** 4-month PFS 51%; median PFS 8.7-months; ORR 9%	[117], [118]
**Surufatinib**	**FGFR1**, CSF1R, VEGFR1/2/3	Phase I/II (NCT05093322)	Paediatric, adolescent, and young adults with relapsed and refractory solid tumours **with known or expected dysfunction in VEGFR1/2/3, CSF1R or FGFR1**	Active, not recruiting	NA
**Ponatinib**	**FGFR1/2/3/4**, CSF1R, PDGFR, VEGFR1/3, RET	Case report	***FGFR3-PHGDH*** oligodendroglioma (10 years)	Partial response, sustained for 7 months at time of reporting	[119]
*FGFR-selective inhibitors*					
**Erdafitinib**	**FGFR1/2/3/4**	NCI-COG- Paediatric MATCH trial (Phase II, NCT03155620)	Paediatric (1–21 years) relapsed or refractory solid tumours, non-Hodgkin lymphoma, or histiocytic disorders **with identified FGFR mutations**	Currently recruiting; response data yet to be reported (*n* = 131 patients enroled in a treatment arm; # in arm B (erdafitinib) not disclosed)	[41]
		RAGNAR study (Phase II, NCT04083976)	Paediatric patients with advanced solid tumours **with FGFR gene alterations** (6-18 years)	Recruiting, response data yet to be reported	[126]
		Case report	***FGFR1-EBF2*** fusion positive-spindle and round cell neoplasm (9-months)	Considerable reduction in tumour size following 12-week treatment (no longer tender to palpation). Treatment ongoing at time of report.	[42]
**Debio1347**	**FGFR1/2/3**	Single centre study (MSKCC) with patients treated under single patient use protocols	Paediatric Patients With Recurrent or Refractory **FGFR-Altered** Gliomas (13-months-14 years)	4/5 patients were evaluable (3 x LGG and 1 x HGG); 2 PR and 2 SD	[124]
**Pemigatinib**	**FGFR1/2/3/4**	Case report (patient enroled in FIGHT-101 (NCT02393248))	**FGFR1 N546K** mutant juvenile pilocytic astrocytoma (32-year-old male; first diagnosed with PA at 13 years of age)	Partial response (91% reduction in tumour size as best response over 18 months of sustained response)	[125]

For studies where a molecular indication was part of the inclusion criteria, the **aberration is bolded**. All other studies were performed on unselected patients.

*PFS* progression free survival, *OS* overall survival, *ORR* overall response rate, *PR* partial response, *MR* minor response, *SD* stable disease.

Multiple kinase inhibitors are also in clinical trial for different subtypes of paediatric brain tumours, but these are based on molecular targets, and the agents themselves are more selective [120]. The potentially efficacy of the MEK inhibitor, trametinib in paediatric LGG patients with MAPK pathway activation (BRAF or NF1 aberrations) has been demonstrated in multiple retrospective studies [121–123], and are now in clinical trial. Given RAS pathway activating mutations co-occur with FGFR alternations in multiple paediatric tumour types, including LGG, trametinib and FGFR inhibitor represents a potential effective therapeutic combination for these patients.

The refinement in both molecular selection of patients, aided by personalised medicine, with FGFR-driven disease has been accompanied with the development of selective FGFR inhibitors. A number of these more selective FGFR inhibitors are in clinical development, including but not limited to erdafitinib, AZD4557, and pemigatinib. Some of these are currently being trialled in paediatric cancer, although little clinical efficacy data is available, and is limited to case reports or small cohort studies (Table 2). A single-centre study of Debio1347 (FGFR1/2/3 inhibitor) in 5 paediatric patients with recurrent or refractory FGFR-altered gliomas demonstrated promising results, inducing partial responses (PR) in two (1 x LGG and 1 x HGG) and stable disease (SD) in two (2 x LGG) of the four evaluable patients [124]. In addition, a partial response was observed in a young adult FGFR1 N546K-mutant pilocytic astrocytoma patient treated with pemigatinib, an FGFR1/2/3/4 ATP-competitive inhibitor [125]. This was an interesting result as the FGFR1 N546K mutation has been shown to mediate resistance to other ATP-competitive inhibitors, as previously described [52]. Erdafitinib is currently being tested in a Phase II clinical trial for treatment of paediatric patients (1–21 years of age) with relapsed or refractory solid tumours, non-Hodgkin lymphoma, or histiocytic disorders with identified FGFR mutations, as part of the NCI-COG Paediatric MATCH trial [41]. In addition, paediatric patients are currently being recruited for the RAGNAR study (NCT04083976) of erdafitinib in advanced solid tumours with FGFR alterations [126]. Outside of these clinical trials, erdafitinib demonstrated some clinical efficacy in a case of *FGFR1-EBF2* positive spindle and round cell neoplasm [42] Notably, infigratinib, an FGFR12/3 selective inhibitor, has also been trialled in adults with *FGFR*-altered recurrent gliomas, where an 6-month PFS rate of 16% and sustained responses of more than a year were observed [127]. Interestingly, 3 of the 4 patients with durable clinical responses harboured FGFR1 K656E (*n* = 2) or the equivalent FGFR3 K650E (*n* = 1) mutations, the only patients with these specific variants in the cohort, while most patients harboured FGFR3 amplifications or FGFR3-TACC3 fusions. At present, erdafitinib and pemigatinib are the only FDA approved FGFR inhibitors, approved for adult advanced urothelial carcinoma and cholangiocarcinoma, respectively.

## Conclusions

*FGFR* genomic alteration, alternate transcript expression, and aberrant *FGF* ligand expression have individually been shown to contribute to FGFR pathway activation in multiple cancer types. While mechanistic studies of these variants in paediatric cancer subtypes is limited, there are numerous studies in adult cancers from which the potential functional impact of these variants can be speculated. The impact of overexpression of *FGFR* RNA in the absence of genomic alteration and the possible cooperation of multiple FGF/FGFR events to drive FGFR pathway activation has not been fully explored or established. The rapid development and advancement of personalised medicine pipelines, to not only include genomic, but now transcriptome analysis, allows for the comprehensive analysis of the vast array of aberrations that can lead to FGFR pathway activation in paediatric cancer. As personalised medicine becomes standard, there is a greater capacity to identify individual patients that are likely to benefit from FGFR-targeted therapies.

## Supplementary information

Supplementary Table 1
