# Supplementary material for: Biological and clinical implications of FGFR aberrations in paediatric and young adult cancers
Source: Oncogene. 2023 May 2;42(23):1875–88. doi: 10.1038/s41388-023-02705-7 (PMC10244177; doi:10.1038/s41388-023-02705-7)
Supplement: Supplementary file 1 — Supplementary Table 1 [file 41388_2023_2705_MOESM1_ESM.docx]

**Supplementary Table 1. Summary of *FGFR* aberrations identified in paediatric and young adult cancers**

| **Gene** | **Type** | **Alteration** | **Cancer type** | **References** |
| --- | --- | --- | --- | --- |
| *FGFR1* | CNA | *FGFR1* amp | RMS | (1-4) |
|  |  |  | OS | (1, 3-6) |
|  |  |  | ES | (4) |
|  |  |  | MPNST | (4) |
|  |  |  | DNET | (7) |
|  |  |  | WT | (7) |
|  |  |  | HGG | (3, 8) |
|  | ITD | *FGFR1* TKD dup | PA | (9) |
|  |  |  | DA | (9-11) |
|  |  |  | OA | (9) |
|  |  |  | DNET | (9-11) |
|  |  |  | ODG | (9-12) |
|  |  |  | GG | (10, 11) |
|  |  |  | LGG, NOS | (10) |
|  |  |  | HGG | (2, 3) |
|  | SNV | N546K | WT | (6, 13) |
|  |  |  | NBL | (6, 13, 14) |
|  |  |  | LGG | (4, 6) |
|  |  |  | OA | (9) |
|  |  |  | ODG | (11, 15) |
|  |  |  | PA | (1, 7, 9) |
|  |  |  | HGG | (2, 3, 6, 15) |
|  |  |  | CNS other | (6) |
|  |  |  | Medulloblastoma | (4) |
|  |  |  | Glioblastoma | (4) |
|  |  |  | Extra-adrenal paraganglioma | (4) |
|  |  | N546D | NBL | (3) |
|  |  |  | HGG | (8) |
|  |  |  | Glioblastoma | (4) |
|  |  | K656E | WT | (13) |
|  |  |  | Medulloblastoma | (6) |
|  |  |  | HGG | (1, 6) |
|  |  |  | LGG | (4, 6) |
|  |  |  | PA | (1) |
|  |  |  | DNET | (4, 7, 11) |
|  |  |  | DA | (6) |
|  |  | K656D | DNET | (11) |
|  |  | K656M | LGG | (6) |
|  |  | K656N | LGG | (4) |
|  |  | R659L | DNET | (11) |
|  |  | K655V | DNET | (11) |
|  |  | D652G | LGG | (4) |
|  |  | I651K | DNET | (11) |
|  |  | H649R | DNET | (11) |
|  |  | D647H | DNET | (11) |
|  |  | D577N | HGG | (8) |
|  |  | M532T | Adrenal cortical carcinoma | (4) |
|  |  | R445W | B-ALL | (13) |
|  |  |  | OS | (3) |
|  |  | V396I | ODG | (11) |
|  |  | V273L | B-ALL | (12) |
|  |  | S251F | RMS | (4) |
|  |  | S302P | Meningioma | (3) |
|  |  | I639V | B-ALL | (12) |
|  |  | N725S | MRT | (3) |
|  | Fusion | *FGFR1-TACC1* | PA | (9, 10) |
|  |  |  | DA | (6, 9-11) |
|  |  |  | OA | (9) |
|  |  |  | DNET | (11) |
|  |  |  | LGG, NOS | (10)**^#^** |
|  |  |  | LGG | (4) |
|  |  |  | HGG | (6) |
|  |  |  | RMS | (6) |
|  |  | *CNTRL-FGFR1* | Myeloid/lymphoid neoplasms with *FGFR1* rearrangement | (16-22) |
|  |  | *ZMYM2-FGFR1* |  | (23-28) |
|  |  | *BCR-FGFR1* |  | (29, 30) |
|  |  | *HOOK3-FGFR1* | B-ALL | (31)**^#^** |
|  |  | *FGFR1-HOOK3* | GIST (STS) | (32) |
|  |  | *FOXO1-FGFR1* | RMS | (33)**^#^** |
|  |  | *FGFR1-ERC1* | HGG | (2) |
|  |  | *FGFR1-EBF2* | Spindle cell sarcoma | (4) |
|  |  | *FGFR1-CLIP2* | LGG | (4) |
|  |  | *WHSC1L1-FGFR1* | OS | (6)**^#^** |
|  | Indel | *FGFR1* indel | DIG | (10)**^#^** |
| *FGFR2* | CNA | *FGFR2* amp | RMS | (3) |
|  |  |  | DNET | (11) |
|  | SNV | P253R | Dysgerminoma | (12) |
|  |  | W290C | Choroid plexus carcinoma | (6) |
|  |  | C382R | WT | (4) |
|  |  | S354C | OS | (4) |
|  |  | R450H | NBL | (3) |
|  |  | R450C | PA | (7) |
|  |  | V512M | ALL | (3) |
|  |  | M535L | NBL | (4) |
|  |  | K659E | OS | (6) |
|  | Fusion | *FGFR2-CTNNA3* | ODG | (10) |
|  |  |  | PLNTY | (34) |
|  |  |  | LGG | (4) |
|  |  | *FGFR2-ERC1* | ODG | (10) |
|  |  | *FGFR2-INA* | ODG | (10) |
|  |  |  | DNET | (7) |
|  |  | *FGFR2-SHTN1* | PLNTY | (34) |
| *FGFR3* | CNA | *FGFR3* amp | Hepatoblastoma | (12) |
|  |  |  | RMS | (3) |
|  |  |  | Ependymoma | (3) |
|  |  |  | Choroid plexus carcinoma | (4) |
|  | SNV | R248C | B-ALL | (13) |
|  |  |  | Hepatoblastoma | (6) |
|  |  | R669Q | WT | (4) |
|  | Fusion | *FGFR3-TACC3* | OA | (9) |
|  |  |  | DA | (11) |
|  |  |  | PLNTY | (34, 35) |
|  |  |  | OS | (3) |
|  |  |  | Papillary urothelial carcinoma | (4) |
|  |  |  | Transitional cell carcinoma | (4) |
| *FGFR4* | CNA | *FGFR4* amp | RMS | (3, 4, 6) |
|  | SNV | N535K | RMS | (1, 4, 6, 36) |
|  |  | N535D | RMS | (3, 36) |
|  |  |  | Sarcoma other | (4) |
|  |  | V550L | RMS | (4, 6, 36) |
|  |  |  | Hepatocellular carcinoma | (4) |
|  |  | V550E | RMS | (4, 36) |
|  |  | A554V | RMS | (36) |
|  |  | G576D | RMS | (36) |
|  |  | Y547C | CNS-PNET | (3) |
|  |  | M504T | Astrocytoma | (4) |
|  |  | R78H | B-ALL | (37) |
|  |  | E475K | Carcinoma | (7) |
|  |  | C56S | Papillary thyroid carcinoma | (7) |
|  |  |  | RMS | (36) |
|  |  | R72L | RMS | (36) |
|  |  | T122A | RMS | (36) |
|  |  | R723H | PA | (7) |
|  |  | A509V | HGG | (7) |
|  |  | G118V | ODG | (7) |
|  |  | I10V | RMS | (36) |
|  |  | L136P | RMS | (36) |
|  |  | A175T | RMS | (36) |
|  |  | R384H | RMS | (36) |
|  |  | V168I | Sarcoma-other | (7) |
| **Abbreviations:** CNA = copy number alteration; ITD = internal tandem duplication; SNV = single nucleotide variant; RMS = rhabdomyosarcoma; amp = amplification; OS = osteosarcoma; MRT = malignant rhabdoid tumour; DNET = dysembrioplastic neuroepithelial tumour; PA = pilocytic astrocytoma; DA = diffuse astrocytoma; OA = oligoastrocytoma; ODG = oligodendroglioma; GG = ganglioglioma; LGG, NOS = low-grade glioma, not otherwise specified; HGG = high-grade glioma; WT = Wilms tumour; NBL = neuroblastoma; B-ALL = B-cell acute lymphoblastic leukaemia; DIG = desmoplastic infantile ganglioglioma; CNS-PNET = CNS primitive neuroectodermal tumour; ES = Ewing sarcoma; MPNST = Malignant Peripheral Nerve Sheath Tumor  **^#^**Genomic structure of the variant not known/not published and as such not included in Figure 4 | | | | |

**References**

1. Gröbner SN, Worst BC, Weischenfeldt J, Buchhalter I, Kleinheinz K, Rudneva VA, et al. The landscape of genomic alterations across childhood cancers. Nature. 2018.

2. Wong M, Mayoh C, Lau LMS, Khuong-Quang D-A, Pinese M, Kumar A, et al. Whole genome, transcriptome and methylome profiling enhances actionable target discovery in high-risk pediatric cancer. Nature Medicine. 2020.

3. van Tilburg CM, Pfaff E, Pajtler KW, Langenberg KPS, Fiesel P, Jones BC, et al. The pediatric precision oncology INFORM registry: clinical outcome and benefit for patients with very high-evidence targets. Cancer Discovery. 2021:candisc.0094.2021.

4. Lazo De La Vega L, Comeau H, Sallan S, Al-Ibraheemi A, Gupta H, Li YY, et al. Rare FGFR Oncogenic Alterations in Sequenced Pediatric Solid and Brain Tumors Suggest FGFR Is a Relevant Molecular Target in Childhood Cancer. JCO Precis Oncol. 2022;6:e2200390.

5. Fernanda Amary M, Ye H, Berisha F, Khatri B, Forbes G, Lehovsky K, et al. Fibroblastic growth factor receptor 1 amplification in osteosarcoma is associated with poor response to neo-adjuvant chemotherapy. Cancer Med. 2014;3(4):980-7.

6. Parsons DW, Janeway KA, Patton DR, Winter CL, Coffey B, Williams PM, et al. Actionable Tumor Alterations and Treatment Protocol Enrollment of Pediatric and Young Adult Patients With Refractory Cancers in the National Cancer Institute-Children's Oncology Group Pediatric MATCH Trial. Journal of Clinical Oncology. 2022;40(20):2224-+.

7. Surrey LF, MacFarland SP, Chang F, Cao K, Rathi KS, Akgumus GT, et al. Clinical utility of custom-designed NGS panel testing in pediatric tumors. Genome medicine. 2019;11(1):32.

8. Harttrampf AC, Lacroix L, Deloger M, Deschamps F, Puget S, Auger N, et al. Molecular Screening for Cancer Treatment Optimization (MOSCATO-01) in Pediatric Patients: A Single-Institutional Prospective Molecular Stratification Trial. Clinical Cancer Research. 2017;23(20):6101.

9. Zhang J, Wu G, Miller CP, Tatevossian RG, Dalton JD, Tang B, et al. Whole-genome sequencing identifies genetic alterations in pediatric low-grade gliomas. Nat Genet. 2013;45(6):602-12.

10. Ryall S, Zapotocky M, Fukuoka K, Nobre L, Guerreiro Stucklin A, Bennett J, et al. Integrated Molecular and Clinical Analysis of 1,000 Pediatric Low-Grade Gliomas. Cancer Cell. 2020;37(4):569-83.e5.

11. Qaddoumi I, Orisme W, Wen J, Santiago T, Gupta K, Dalton JD, et al. Genetic alterations in uncommon low-grade neuroepithelial tumors: BRAF, FGFR1, and MYB mutations occur at high frequency and align with morphology. Acta Neuropathol. 2016;131(6):833-45.

12. Newman S, Nakitandwe J, Kesserwan CA, Azzato EM, Wheeler DA, Rusch M, et al. Genomes for Kids: The scope of pathogenic mutations in pediatric cancer revealed by comprehensive DNA and RNA sequencing. Cancer Discovery. 2021:candisc.1631.2020.

13. Ma X, Liu Y, Liu Y, Alexandrov LB, Edmonson MN, Gawad C, et al. Pan-cancer genome and transcriptome analyses of 1,699 paediatric leukaemias and solid tumours. Nature. 2018.

14. Brady SW, Liu Y, Ma X, Gout AM, Hagiwara K, Zhou X, et al. Pan-neuroblastoma analysis reveals age- and signature-associated driver alterations. Nat Commun. 2020;11(1):5183.

15. Wu G, Diaz AK, Paugh BS, Rankin SL, Ju BS, Li YJ, et al. The genomic landscape of diffuse intrinsic pontine glioma and pediatric non-brainstem high-grade glioma. Nature genetics. 2014;46(5):444-50.

16. Sarthy JF, Reddivalla N, Radhi M, Chastain K. Pediatric 8p11 eosinophilic myeloproliferative syndrome (EMS): A case report and review of the literature. Pediatric blood & cancer. 2017;64(5).

17. Brown LM, Bartolo RC, Davidson NM, Schmidt B, Brooks I, Challis J, et al. Targeted therapy and disease monitoring in CNTRL-FGFR1-driven leukaemia. Pediatric blood & cancer. 2019;66(10):e27897.

18. Hu S, He Y, Zhu X, Li J, He H. Myeloproliferative disorders with t(8;9)(p12;q33): a case report and review of the literature. Pediatric hematology and oncology. 2011;28(2):140-6.

19. Sohal J, Chase A, Mould S, Corcoran M, Oscier D, Iqbal S, et al. Identification of four new translocations involving FGFR1 in myeloid disorders. Genes Chromosomes Cancer. 2001;32(2):155-63.

20. Nakayama H, Inamitsu T, Ohga S, Kai T, Suda M, Matsuzaki A, et al. Chronic myelomonocytic leukaemia with t(8;9)(p11;q34) in childhood: an example of the 8p11 myeloproliferative disorder? British journal of haematology. 1996;92(3):692-5.

21. Van Den Berg H, Kroes W, van der Schoot CE, Dee R, Pals ST, Bouts TH, et al. A young child with acquired t(8;9)(p11;q34): additional proof that 8p11 is involved in mixed myeloid/T lymphoid malignancies. Leukemia. 1996;10(7):1252-3.

22. Lewis JP, Jenks H, Lazerson J. PHILADELPHIA CHROMOSOME-NEGATIVE CHRONIC MYELOGENOUS LEUKEMIA IN A CHILD WITH T(8-9)(P11 OR 12-Q34). American Journal of Pediatric Hematology Oncology. 1983;5(3):265-9.

23. Zhang WW, Habeebu S, Sheehan AM, Naeem R, Hernandez VS, Dreyer ZE, et al. Molecular monitoring of 8p11 myeloproliferative syndrome in an infant. J Pediatr Hematol Oncol. 2009;31(11):879-83.

24. Kuskonmaz B, Kafali C, Akcoren Z, Karabulut HG, Akalin I, Tuncer MA. The 8p11 myeloproliferative syndrome in a 3-year-old child. Leukemia research. 2008;32(1):198-9.

25. Wong WS, Cheng KC, Lau KM, Chan NP, Shing MM, Cheng SH, et al. Clonal evolution of 8p11 stem cell syndrome in a 14-year-old Chinese boy: a review of literature of t(8;13) associated myeloproliferative diseases. Leuk Res. 2007;31(2):235-8.

26. Somers GR, Slater H, Rockman S, Ekert H, Southey MC, Chow CW, et al. Coexistent T-cell lymphoblastic lymphoma and an atypical myeloproliferative disorder associated with t(8;13)(p21;q14). Pediatric Pathology & Laboratory Medicine. 1997;17(1):141-58.

27. Inhorn RC, Aster JC, Roach SA, Slapak CA, Soiffer R, Tantravahi R, et al. A syndrome of lymphoblastic lymphoma, eosinophilia, and myeloid hyperplasia/malignancy associated with t(8;13)(p11;q11): description of a distinctive clinicopathologic entity. Blood. 1995;85(7):1881-7.

28. Abruzzo LV, Jaffe ES, Cotelingam JD, Whang-Peng J, Del Duca V, Jr., Medeiros LJ. T-cell lymphoblastic lymphoma with eosinophilia associated with subsequent myeloid malignancy. Am J Surg Pathol. 1992;16(3):236-45.

29. Dolan M, Cioc A, Cross NC, Neglia JP, Tolar J. Favorable outcome of allogeneic hematopoietic cell transplantation for 8p11 myeloproliferative syndrome associated with BCR-FGFR1 gene fusion. Pediatr Blood Cancer. 2012;59(1):194-6.

30. Haslam K, Langabeer SE, Kelly J, Coen N, O'Connell NM, Conneally E. Allogeneic Hematopoietic Stem Cell Transplantation for a BCR-FGFR1 Myeloproliferative Neoplasm Presenting as Acute Lymphoblastic Leukemia. Case reports in hematology. 2012.

31. Reshmi SC, Harvey RC, Roberts KG, Stonerock E, Smith A, Jenkins H, et al. Targetable kinase gene fusions in high-risk B-ALL: a study from the Children's Oncology Group. Blood. 2017;129(25):3352-61.

32. Shi E, Chmielecki J, Tang CM, Wang K, Heinrich MC, Kang G, et al. FGFR1 and NTRK3 actionable alterations in "Wild-Type" gastrointestinal stromal tumors. J Transl Med. 2016;14(1):339.

33. Liu J, Guzman MA, Pezanowski D, Patel D, Hauptman J, Keisling M, et al. FOXO1-FGFR1 fusion and amplification in a solid variant of alveolar rhabdomyosarcoma. Mod Pathol. 2011;24(10):1327-35.

34. Huse JT, Snuderl M, Jones DTW, Brathwaite CD, Altman N, Lavi E, et al. Polymorphous low-grade neuroepithelial tumor of the young (PLNTY): an epileptogenic neoplasm with oligodendroglioma-like components, aberrant CD34 expression, and genetic alterations involving the MAP kinase pathway. Acta neuropathologica. 2017;133(3):417-29.

35. Chen Y, Tian T, Guo X, Zhang F, Fan M, Jin H, et al. Polymorphous low-grade neuroepithelial tumor of the young: case report and review focus on the radiological features and genetic alterations. BMC Neurology. 2020;20(1):123.

36. Taylor JGt, Cheuk AT, Tsang PS, Chung JY, Song YK, Desai K, et al. Identification of FGFR4-activating mutations in human rhabdomyosarcomas that promote metastasis in xenotransplanted models. J Clin Invest. 2009;119(11):3395-407.

37. Gu Z, Churchman ML, Roberts KG, Moore I, Zhou X, Nakitandwe J, et al. PAX5-driven subtypes of B-progenitor acute lymphoblastic leukemia. Nat Genet. 2019;51(2):296-307.
